# Evaluation of a Web-Based ADHD Awareness Training in Primary Care: Pilot Randomized Controlled Trial With Nested Interviews

**DOI:** 10.2196/19871

**Published:** 2020-12-11

**Authors:** Blandine French, Charlotte Hall, Elvira Perez Vallejos, Kapil Sayal, David Daley

**Affiliations:** 1 Institute of Mental Health, School of Medicine University of Nottingham Nottingham United Kingdom; 2 Division of Psychiatry & Applied Psychology School of Medicine University of Nottingham Nottingham United Kingdom

**Keywords:** ADHD, primary care, general practice, randomized controlled trial, online intervention, interviews

## Abstract

**Background:**

Attention-deficit/hyperactivity disorder (ADHD) is a neurodevelopmental disorder affecting up to 5% of children and adults. Undiagnosed and untreated ADHD can result in adverse long-term health, educational, and social impacts for affected individuals. Therefore, it is important to identify this disorder as early as possible. General practitioners (GPs) frequently play a gatekeeper role in access to specialist services in charge of diagnosis and treatment. Studies have shown that their lack of knowledge and understanding about ADHD can create barriers to care.

**Objective:**

This pilot randomized controlled trial assesses the efficacy of a web-based psychoeducation program on ADHD tailored for GPs.

**Methods:**

A total of 221 participants were randomized to either a sham intervention control or an awareness training intervention and they completed questionnaires on ADHD knowledge, confidence, and attitude at 3 time points (preintervention, postintervention, and 2-week follow-up). Participants in the intervention arm were invited to participate in a survey and follow-up interview between 3 and 6 months after the intervention.

**Results:**

The responses of 109 GPs were included in the analysis. The knowledge (*P*<.001) and confidence (*P*<.001) of the GPs increased after the intervention, whereas misconceptions decreased (*P*=.04); this was maintained at the 2-week follow-up (knowledge, *P*<.001; confidence, *P*<.001; misconceptions, P=.03). Interviews and surveys also confirmed a change in practice over time.

**Conclusions:**

These findings demonstrate that a short web-based intervention can increase GPs’ understanding, attitude, and practice toward ADHD, potentially improving patients’ access to care.

**Trial Registration:**

International Standard Randomized Controlled Trial Number ISRCTN45400501; http://www.isrctn.com/ISRCTN45400501.

## Introduction

### Background

Attention-deficit/hyperactivity disorder (ADHD) is a neurodevelopmental disorder that affects up to 5% of children and adults [1]. The symptoms experienced by individuals with ADHD lead to considerable behavioral and cognitive impairment [2,3]. In adulthood, risks associated with undiagnosed and untreated ADHD, such as relationship or employment difficulties, can strongly affect the mental health of individuals and lead to economic and social burden [4]. Gaining a diagnosis of ADHD is important for access to appropriate treatment and minimizing the long-term impacts of ADHD. However, in many countries, ADHD is underdiagnosed and undertreated [5-7]. For example, in the United Kingdom, figures show that ADHD is underdiagnosed and undertreated, with 0.73% of children and 0.06% of adults receiving ADHD medication [8].

In the United Kingdom, general practitioners (GPs) often act as gatekeepers to specialist services where diagnosis and treatment take place. GPs do not always readily recognize ADHD symptoms, with many reporting low confidence, limited knowledge, and strong misconceptions about the disorder [9-11]. This is a key barrier for individuals with ADHD in accessing care [10]. Therefore, the development of interventions targeted at increasing the knowledge and confidence of the GPs is essential.

GPs in the United Kingdom need to participate in ongoing continuing professional development (CPD) to keep up to date with medical knowledge and changes in practice. Although many training packages and programs are continually being developed to improve medical skills of GPs [12-16], to our knowledge, there are no current web-based programs aimed at ADHD. Some published evidence indicates that primary care training on specific topics can improve patient care [15,17,18]; clinical outcomes [17]; and GP knowledge, confidence, and attitudes [19-21], highlighting the potential benefit for a targeted ADHD education package.

One perceived barrier to GPs attending and participating in training may be having to travel long distances to attend training sessions, which may be particularly burdensome for GPs serving in remote communities [22]. The development of web-based training may turn out to be beneficial in reducing this barrier, offering GPs easily accessible training at a time and place that fits their busy schedules. The use of web-based training by health care professionals has significantly increased in recent years [23-25]. Web-based training is an efficacious mode of delivery, with a recent review demonstrating that web-based continuing medical education improves knowledge and changes GPs’ practice [22]. To our knowledge, no studies have been published on ADHD web-based psychoeducation programs developed for GPs, and data on the efficacy of ADHD training programs for GPs are lacking.

### Objectives

This study presents the evaluation of a web-based intervention for GPs on ADHD. The web-based intervention was developed by the researchers following a strict development process, and its usability has been previously assessed [26]. In line with the Medical Research Council recommendations on the development and evaluation of complex interventions [27], this study aims to obtain preliminary findings on the effect of the *understanding ADHD in primary care* web-based program on the ADHD knowledge, attitudes, misconceptions, and change of practice of GPs to determine whether a future definitive randomized controlled trial (RCT) should be conducted. GP participants’ opinions on the intervention and perceived impact on practice were obtained via qualitative interviews and a postintervention survey.

## Methods

### Study Design

The *understanding ADHD in primary care* trial was a pilot RCT registered with the International Standard Randomized Controlled Trial Number (ISRCTN) registry (ISRCTN45400501), with nested qualitative interviews. This parallel-group, single-blind RCT was conducted between August and November 2019 in primary care services in England. The interviews took place after the intervention between December 2019 and March 2020. The study received ethical approval from the University of Nottingham, Faculty of Medicine and Health Sciences Research Ethics Committee (reference: 19/HRA/1028; February 20, 2019) and from the Nottinghamshire Healthcare National Health Service (NHS) Foundation Trust Research and Development department (project ID 257567).

### Participants

GPs and GP trainees were recruited from multiple sites across England, and they responded to invitation emails from local clinical research networks (CRNs) sent out via their practice. A total of 12 out of 15 English CRNs distributed the study invitation to hundreds of practices. Interested GP practices then circulated the study details to their GPs and trainees, with instructions to contact the lead researcher to express interest in the study. GPs and GP trainees practicing in England were included; the only exclusion criterion was having taken part in a previous usability study. Participants who expressed interest were sent a link to a web-based consent form. Multiple expressions of interest were received, representing most of England, and 231 consent forms were signed over 2 weeks. Unfortunately, it is not possible to know the exact number of expressions of interest; we received over 500 emails and were not able to map the sites that signed up after initial contact with us. Written informed consent was obtained for each participant before taking part in the study. Participants from the control group were sent a link to the web-based course after taking part in the study. Participants from the intervention group were invited to participate in a short qualitative interview and survey after completion of the intervention. Participants received an inconvenience allowance for participating in the study.

### Intervention

#### Intervention: Understanding of ADHD in a Primary Care Web-Based Resource

The web-based noncommercial resource was delivered using a University of Nottingham server and built with an open-source learning management system. Further details on intervention development are reported in the study by French et al [26]. The complete web-based resource consisted of two 20-minute modules undertaken sequentially. The 2 modules followed the same format with text on the left side of the screen and interactive activities on the right. The activities included patient testimonies, drag and drop games, specialist videos, and pictures.

The 2 modules of the web-based resource are as follows:

- Module 1, called understanding ADHD, included the heterogeneous nature of ADHD; a brief description of ADHD epidemiology and neuroscience; and ADHD symptoms, comorbidity, risks, and common misconceptions.
- Module 2, called the role of the GP, introduced the role of the GP in ADHD diagnosis and treatment pathways; the identification of ADHD and subsequent treatment options; the gatekeeping role of the GP and the pathway to care in the United Kingdom; and an ADHD toolkit, including downloadable screening tools, strategies, or useful websites [28].

#### Control Web-Based Resource

Participants allocated to the sham control group watched a web-based 30-minute video about the University of Nottingham Institute of Mental Health [29]. No information related to ADHD was provided during this video.

No changes were made to either the interventions or control during the trial.

### Measures and Outcomes

#### Pilot RCT

##### Demographic Questionnaire

Exploration of demographic variables included the impact of the demographics of the participants on the Knowledge of Attention Deficit Disorders Scale (KADDS) scores. The demographics of the participants were recorded through a brief questionnaire developed by the study team at baseline (time point 1 [T1]).

##### Primary Outcome

The primary outcome was a change in the knowledge of the GPs assessed by the KADDS [30] questionnaire scores (T1 to time point 2 [T2] which is the primary end point). The knowledge of the participants was assessed using an adapted version of the KADDS and the GPs’ understanding of the ADHD questionnaire [31].

##### KADDS Questionnaire

This 39-item self-report scale was originally developed to measure the understanding and knowledge of ADHD among teachers [32]. However, the itemized questions were not solely relevant to teachers and were also pertinent to general knowledge and the understanding of ADHD among GPs. A total of 27 questions from this questionnaire were used in this evaluation.

##### Secondary Outcomes

Changes in knowledge (assessed via the KADDS questionnaire) were reassessed 2 weeks after completing the intervention (time point 3 [T3]). The subscales of the KADDS questionnaire were also analyzed. Further secondary outcomes included the confidence of GPs in ADHD, awareness among GPs of the ADHD questionnaire, and usability questionnaire.

###### Confidence of GPs in ADHD

Change in confidence was explored through a self-rated visual analog scale (1=low and 10=high) assessing the confidence of GPs in their knowledge of ADHD.

###### Awareness of GPs of the ADHD Questionnaire

This questionnaire assesses the attitudes of GPs toward and their experience of ADHD [31]. Some questions were excluded as they were not relevant to the British health care system or were similar to the ones asked by the KADDS. A total of 13 questions from this questionnaire were used as they were specifically tailored to the experiences of GPs.

These questionnaires were administered on the web at 3 time points: baseline (T1), immediately after taking part in the study (T2), and 2 weeks after completing the study (T3). The time window for T3 was 2 weeks (−3 days or +10 days). The questions were the same at all time points and for both groups.

###### Usability Questionnaire

Participants in the intervention arm completed 2 visual analog scales on the usefulness of the intervention information and the likely impact on their practice at T2 only.

#### Postintervention Interviews and Survey

A 4-item open questionnaire was sent to all 56 participants from the intervention arm who consented to assess changes in practice and approaches 6 months after the intervention.

Secondary outcomes also included exploration of attitudes toward ADHD and long-term self-reported change in practice. Changes in practice were assessed through semistructured interviews and a short survey. The interview schedule included questions about the intervention and the impact it had on the attitude and practice of the GPs. As the aim of the interviews was to gauge the change in practice, it was noticed that 3 months was a short time to effectively assess this. Therefore, after conducting 11 interviews, it was decided that the remaining 12 interviews will be conducted 6 months after the intervention.

The outcome assessor and interviewer were not blinded to group allocation.

### Randomization

Once recruited, participants were randomized before baseline data collection into either the intervention or the control arm. Randomization was initiated by the primary author and performed on the web through a randomization website [33] in batches of 20. Owing to the nature of the study, participants were blind to the study arm but may have been able to guess their arm once they started the study.

### Procedures

Details of the study were sent to practices that had registered an interest in research within local CRNs. Participants wishing to participate signed a web-based consent form. Upon receiving consent, they were randomly allocated to the intervention or control group. After randomization, participants were then sent a link to the web-based resource of their allocated group. Upon following the link, both groups were directed to complete the baseline questionnaires (T1). After completion, an external link at the end of the questionnaire directed the GPs to their allocated intervention (ie, intervention or control). Upon completion of the intervention, both groups completed immediate follow-up measures (T2). Weekly reminders were sent via email for 4 weeks by the researcher. Follow-up measures were completed again 2 weeks postintervention (T3). All elements of the intervention were compulsory, and participants had to take part in all stages to contribute to the study. An inconvenience allowance and CPD certificate from the Royal College of GPs (RCGP) were attributed to the participants upon completion of the questionnaire at T3.

At 3 and 6 months after taking part, participants who had been allocated to the intervention group and had given consent to be contacted again were asked to take part in follow-up interviews. All interviews were originally planned to be conducted at 3 months (10 interviews); however, after noticing that this timeframe was not long enough, the remaining interviews (13 interviews) were conducted at 6 months. Participants who responded were interviewed over the phone for 15 minutes at the time of their convenience. Semistructured qualitative interviews were conducted over the phone. All 56 participants from the intervention arm who had given consent were also sent a short final survey to complete on the web.

### Data Analysis

#### Data Preparation

##### Protocol Violation

Participants who took longer than 48 hours to complete the first 2 questionnaires were excluded from the analyses, as it was not possible to gauge whether any changes in scores were because of the intervention or external factors. Participants who did not complete all time points were also excluded from the completer analysis as an intention-to-treat analysis was not possible because randomization was done before baseline.

The KADDS questionnaire generated 3 types of responses: *true*, *false*, or *don*’*t know*. These responses were classified into 3 categories: knowledge, misconception, and confidence [32].

- Knowledge included responses that were the right answers. If participants responded correctly to the question, they gained an extra knowledge point.
- Misconceptions included responses that were wrong. If participants responded incorrectly, then their misconception score increased by 1.
- Confidence included responses of don’t know. By not committing to an answer, the lack-of-confidence scores of the participants increased by one.

#### Intervention Analyses Strategy

Preliminary checks were conducted to ensure that there was no violation of the assumptions of normality, linearity, homogeneity of variances, and reliable measurement of the covariate. A significant Kolmogorov-Smirnov test showed that the data were not normally distributed; therefore, nonparametric tests were used. Mann-Whitney U and Kruskal-Wallis tests were used to explore demographic differences between trial arms. A Spearman correlation was used to determine the relationship between the KADDS and confidence scores. The KADDS questionnaire scores were the primary outcome at T2; self-ratings of confidence were also explored; and both variables were analyzed using analyses of covariance (ANCOVAs), with T1 entered as the covariate, as an ANCOVA is robust to violation of the nonparametric assumption with moderate-to-large sample sizes greater than 15 cases per cell [34]. The outcome at T3 was also explored using the same analytical approach. Both the total and subscale scores of the KADDS were explored.

#### Qualitative Interview Analyses and Survey

The analytic strategy for this study was based on thematic analysis [35] enhanced by the principles of grounded theory [36]. Themes and subthemes were identified using an adapted approach of the 6-stage process of Braun and Clarke [20]. The analytic process began by transcribing each interview verbatim shortly after being conducted. Following this process, the lead investigator first familiarized herself with the interviews and made notes in a diary of preliminary thoughts on the content of the interviews. From this, preliminary codes were identified in a coding manual that were then collated and combined to be classified into broader themes using constant comparative analysis, both within and between transcripts. Finally, as the analysis evolved, these broader themes were reviewed and refined to generate the final themes proposed. An ongoing analysis allowed for a clear definition of the final themes.

Themes were finally reviewed by a second researcher (EV) to ensure that they mapped to the original transcripts. Interrater reliability was tested on a small proportion (5/23, 20% of interviews) of the themes of the transcripts. The results were validated collectively as a team, and any discrepancies were discussed and reconciled. The survey responses were reported descriptively and were used to triangulate the responses from the interviews.

## Results

### Pilot RCT

**Figure 1:**
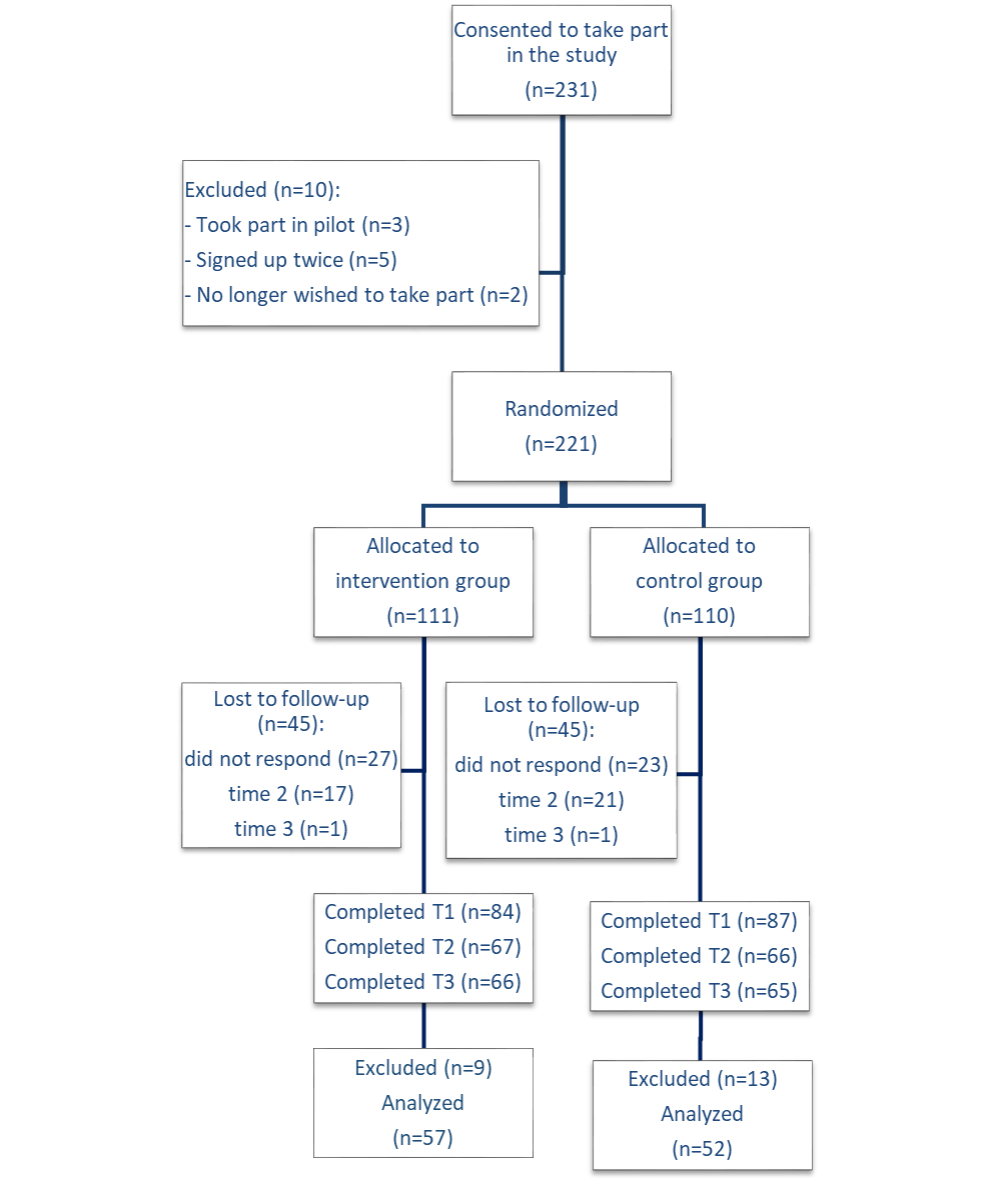
CONSORT (Consolidated Standards of Reporting Trials) flowchart of the pilot randomized controlled trial. A total of 18 participants were excluded because they did not complete the questionnaires at time point 1 (preintervention) and time point 2 (postintervention) within 48 hours, and 4 participants were excluded in the control arm for having received a link to the intervention before completion. T1: time point 1; T2: time point 2; T3: time point 3.

Participants were recruited between July 10 and August 23, 2019 and were followed up until October 30, 2019. When the trial ended, a total of 231 GPs registered their interest in the study and consented to participate. A total of 10 GPs did not meet the eligibility criteria (Figure 1) and were not enrolled in the trial.

Therefore, 221 participants were randomized, 111 in the intervention group and 110 in the control group. After randomization, 51 GPs (27 in the intervention and 23 in the control groups) did not respond to the invitation to start the study. Figure 1 shows the number of participants lost to follow up at each point. Upon answering the baseline questionnaire, 37 GPs did not complete the postquestionnaires at T2 (17 in the intervention and 20 in the control groups) and 2 GPs at T3 (1 in the intervention and 1 in the control group). A total of 170 trainees or fully qualified GPs (103/170, 60.6% female; 6/170, 3.5% GP trainees) completed T1, 133 completed T1 and T2 (84/133, 63.2% female; 5/133, 3.8% GP trainees), and 131 (82/131, 62.6% female; 5/131, 3.8% GP trainees) completed the assessments at all 3 time points.

A total of 22 participants were excluded from the analyses following protocol violations. A total of 18 were excluded as they took longer than 48 hours to complete pre- and postquestionnaires (T1 and T2), and 4 participants from the control group were excluded after T2 as they inadvertently received a link to the intervention before T3.

Figure 1 shows that both trial arms had an even number of recruitments and comparable levels of nonengagement, dropouts, and excluded participants.

### Baseline Characteristics

The baseline characteristics of the study group are summarized in Table 1. Most participants were females (103/170, 60.6%). The age range was fairly, evenly split across the age groups, but most participants were aged below 45 years. However, the range of years of practice was very broad. The estimated number of children under their care with suspected ADHD ranged widely from 0 to 100. The number of individuals diagnosed with ADHD also varied widely. The number of times the participants identified ADHD in their patients was also varied, with most participants reporting that they had not identified more than 5 patients. When asked whether ADHD was part of their medical training, most GPs reported that it was not.

**Table 1:** Baseline characteristics.

Participants		Baseline (n=170)^a^		Participants included in completer analyses (n=109)^a^	
		Control (n=87)	Intervention (n=83)	Control (n=52)	Intervention (n=57)
**Gender, n (%)**					
	Male	29 (33)	38 (46)	15 (28)	27 (47)
	Female	58 (66)	45 (54)	37 (71)	30 (52)
**Age (years), n (%)**					
	25-35	26 (30)	23 (28)	15 (29)	16 (28)
	36-45	34 (39)	29 (35)	20 (38)	21 (37)
	46-55	22 (25)	24 (29)	13 (25)	14 (25)
	56-65	5 (5)	7 (8)	2 (8)	6 (10)
**ADHD^b^ part of general practitioner training, n (%)**					
	Yes	17 (19)	18 (21)	12 (23)	15 (26)
	No	57 (66)	52 (63)	34 (65)	35 (61)
	Unsure	5 (5)	4 (6)	2 (3.8)	1 (2)
	Small part of teaching	8 (10)	8 (10)	4 (7.6)	6 (11)
**Estimated number of children with suspected ADHD seen in practice annually**					
	Mean	14	19	11	16
	Range	0-100	1-150	0-90	1-100
**Individuals with a confirmed ADHD diagnosis currently in practice**					
	Mean	43	67	39	57
	Range	0-400	2-500	0-400	0-500
**Number of times ADHD was picked up by participant**					
	Mean	4.1	5.4	3.2	5.1
	Range	0-30	0-50	0-30	0-50
**Medical experience (years)**					
	Mean	15.1	16.5	14.7	15.9
	Range	0-36	0-36	0-33	0-36

^a^Some data are missing for some questions.

^b^ADHD: attention-deficit/hyperactivity disorder.

### Demographics

The demographics of the participants are presented in Multimedia Appendix 1.

### Study Interaction

Participants were instructed to complete assessments in one go if possible; however, they had the option to log off and return if required. Participants who took longer than 48 hours between T1 and T2 were excluded from the analyses. Participants from the control group mostly completed T1 and T2 in 1 session (41/52, 78%), whereas fewer participants in the intervention group completed T1 and T2 in 1 session (35/57, 61%). Among those who completed T1 and T2 in 1 session, the average time spent on the control video was 39 minutes (SD 20.79; range 13-85), and the average time spent on the intervention course was 55 minutes (SD 13.5; range 28-125). Most participants interacted with the video or intervention in both groups, suggesting that they were unsure of their group allocation.

### Primary Outcome

The primary outcomes for this intervention were KADDS knowledge scores at T2. Table 2 illustrates the responses from these scores and responses from KADDS scores assessed as secondary outcomes.

**Table 2:** Descriptive statistics of the knowledge, misconceptions, and confidence scores of the Knowledge of Attention Deficit Disorders Scale (KADDS) for the 2 groups at the 3 different time points.

Groups		KADDS knowledge^a^	KADDS misconceptions^a^	KADDS confidence^a^	Self-rated confidence^a^
**Control group, mean (SD)**					
	T1^b^	16.82 (5.15)	1.82 (1.78)	7.15 (6.07)	4.40 (1.66)
	T2^c^	17.23 (5.18)	2.05 (1.62)	6.64(5.99)	4.57 (1.67)
	T3^d^	17.13 (5.02)	2.24 (1.77)	6.69 (5.97)	4.88 (1.72)
**Intervention group, mean (SD)**					
	T1	16.65 (3.88)	2.16 (2.20)	7.12 (4.30)	4.66 (1.70)
	T2	23.71 (2.00)	1.54 (1.55)	0.73 (1.35)	7.40 (1.05)
	T3	22.96 (2.13)	1.70 (1.65)	1.22 (1.71)	7.36 (0.89)

aThe knowledge scores of Knowledge of Attention Deficit Disorders Scale (KADDS) represent the number of right answers, KADDS misconception scores represent the number of wrong answers, and KADDS confidence scores represent the number of don’t know answers.

^b^T1: time point 1.

^c^T2: time point 2.

^d^T3: time point 3.

A one-way between-group ANCOVA was conducted to compare the effectiveness of the web-based intervention designed to change the attitudes of the GPs toward ADHD. There was a significant impact of the intervention on ADHD knowledge after controlling for baseline responses, with the intervention group reporting significantly more knowledge of ADHD, *F*_1,106_=117.5, *P*<.001, and partial eta squared=0.52.

In addition, enhanced knowledge from the KADDS questionnaire was retained at the 2-week follow-up, *F*_1,106_=96.25, *P*<.001, and partial eta squared=0.47.

### Secondary Outcomes

#### ADHD Knowledge, Misconceptions, and Confidence

After controlling for differences in baseline responses, the intervention group showed a significant reduction in ADHD misconceptions compared with the control group, *F*_1,106_=4.20, *P*=.04, and partial eta squared=0.03.

This effect was retained at the 2-week follow-up, *F*_1,106_=9.21, *P*=.03, and partial eta squared=0.04.

Immediately after the intervention (T2), the intervention group also showed a significant increase in confidence compared with the control group: *F*_1,106_=182.8, *P*<.001, and partial eta squared=0.63.

This increased confidence was retained at the 2-week follow-up: *F*_1,106_=110.08, *P*<.001, and partial eta squared=0.50.

#### Factor Subscales

The original KADDS questionnaire had 3 subscales: associated features (general information about the nature, causes, and prognosis of ADHD), symptom or diagnosis, and treatment. These subscales aim to reflect content areas relevant to diagnostic decisions. The results of KADDS knowledge scores on these subscales were further explored. Multimedia Appendix 2 presents the responses for each subscale.

For participants in the intervention group, scores decreased on all the subscales after the intervention at T2 and T3—associated features subscale, T2: *F*_1,106_=88, *P*<.001, partial eta squared=0.45 and T3: *F*_1,106_=69, *P*<.001, partial eta squared=0.39; the symptoms/diagnosis subscale, T2: *F*_1,106_=69.8, *P*<.001, partial eta squared=0.39 and T3: *F*_1,106_=57.9, *P*<.001, partial eta squared=0.35; and treatment subscale, T2: *F*_1,106_=45, *P*<.001, partial eta squared=0.30 and T3: *F*_1,106_=45.9, *P*<.001, partial eta squared=0.30.

The relationship between the KADDS knowledge scores at T1 and self-rated confidence was investigated using Spearman rho correlations. A strong positive correlation between the 2 variables was observed, r=0.473, n=109, and *P*<.001, with high levels of self-rated confidence associated with higher scores of ADHD knowledge.

#### Intervention Group

At T2, participants in the intervention group were asked to rate 2 feedback questions on the usefulness of the information and likelihood to inform practice on a scale of 1 to 10. The results indicated that participants found the information to be useful (mean 8.2, SD 1.48) and likely to inform practice (mean 7.8, SD 1.5).

#### Attitude Toward ADHD

Another questionnaire on GPs’ attitudes toward ADHD was included at all time points. Descriptive statistics for these 12 questions are presented in Multimedia Appendix 3.

The findings from this questionnaire demonstrate that most GPs do not endorse most common misconceptions and nonscientific associations with ADHD. However, changes in attitude and these misconceptions can be observed among participants from the intervention group, whereas control participants’ scores remained unchanged over the 3 time points. The slight changes in attitude in the intervention group were mostly related to the following statements:

Most children with ADHD try to control themselves.

Parents seek ADHD diagnosis as an excuse for their child’s bad behavior.

ADHD diagnosis relieves families from stress and supports problem solving.

Do you believe ADHD is society’s excuse for badly behaved children?

### Interviews and Survey

A total of 56 participants who took part in the intervention arm had given consent to be contacted again and were invited to take part in a short qualitative interview and a short survey. A total of 23 participants took part in the interviews, and 21 responded to a brief survey about the impact of the intervention on their clinical practice. The interviews lasted for an average of 10 minutes 30 seconds (range 6.43-15.45).

No differences were observed in the interviews of GPs who took part in the first wave of interviews (3 months) and the second wave of interviews (6 months). GPs reported similar changes in knowledge and practice; however, by allowing more time, a greater impact on practice was observed (more GPs reporting it), allowing training to filter through to their practice.

#### Interviews

##### Feedback on the Intervention

All participants thought that the format of the intervention was informative, useful, and appropriate. None of the participants thought that any content was missing. A couple of participants expressed that there was too much text and that the content could be more concise. Participants benefited mainly from the videos, information about adults, and the genetic explanation of ADHD. Participants highlighted the benefit of understanding the epidemiology and long-term aspects of ADHD as well as having experts’ and patients’ videos to help put ADHD into context, especially the videos of a GP with ADHD.

Participants were also asked about their reasons for signing up for the study. Although monetary rewards and demands to take part in research were cited as incentives, the main incentive was professional/personal interest in the topic. Most GPs stipulated that personal interest in ADHD was the reason they signed up, often acknowledging a lack of previous knowledge and/or medical school training on the topic.

The interviews highlighted 2 main themes, both related to the impact of the intervention. The first theme related to the personal impact the intervention had on the participants, exploring changes in their understanding, attitudes, and knowledge. The second theme explored broader changes and the impact the intervention had on other individuals. This included not only participants’ change in practice, directly impacting their patients, but also the impact the intervention had on their personal lives and broader professional views.

##### Personal Impact: Change in Knowledge and Attitude of GPs

Increased knowledge and attitudes was the first theme highlighted. Most participants reported that taking part in the study greatly increased their knowledge of ADHD, especially as they had received very limited medical training on ADHD. Participants stated that it helped reduce misconceptions and demystified ADHD, which was especially useful for younger GPs or trainees. Many participants found that they knew very little about the topic, specifically with regard to adult diagnosis and biological/genetic components, as many believed or were taught that ADHD was a behavioral problem only present in childhood. Increasing accurate knowledge was especially helpful for GPs as they enjoyed learning about the positives of gaining a diagnosis and accessing the right treatment:

I was surprised how little I knew about it beforehand to be honest… I am much more sympathetic… The fact that I can remember so much about it is probably testament to how good it was at reinforcing and retaining the information.

P12

Participants who had some preliminary knowledge of ADHD stated that the course was a good refresher and confirmed what they already knew while adding a few extra unknown facts. These participants often mentioned that their knowledge was acquired in informal ways throughout their practice, and they felt reassured that this knowledge was confirmed by the intervention. However, a few participants raised the issue that although the intervention was informative, it was too simplistic for individuals who had extensive previous knowledge:

You pick up bits and pieces along the way and I think most of those were covered in the program and then I reckon about 50% I wasn’t aware of.

P9

Increased knowledge and information received from the course led to almost all the participants reporting a change in attitude toward ADHD. More specifically, participants reported feeling more confident and being more understanding and more empathetic toward ADHD. Participants also reported being more tolerant and patient toward people seeking a diagnosis, having less prejudice, and being less dismissive. By demystifying some of the stigmas about ADHD, the resource allowed participants to gain a more empathic approach toward the disorder and change their mindset:

Actually it has changed my attitude, it’s not very often that some sort of learning will do that because attitudes are quite hard engrained.

P1

I’ve got a couple of adults with ADHD (who have been refereed) and I’m able to empathize with them a lot more whilst we are “holding them” until they get to the top of the list to see a psychiatrist.

P19

##### Broader Impact: Change in Practice and Beyond

The second theme these interviews highlighted referred to the broader impacts of the intervention. Many participants reported changing their practice in many different ways. Some reported an increase in identification and referral, acknowledging that the course enabled them to make the process easier and quicker and develop a more structured approach to referrals. Others reported changes in practice in relation to the tools and information that they now use to refer to and manage ADHD, increasing referral to services and screening questionnaires. As one GP mentioned, “It is not so much *what* I do that has changed but *how* I do it.” Some of the knowledge gained, for instance, in relation to the association between ADHD and depression or greater awareness about symptoms in adulthood, has helped GPs to now explore patients’ histories further and ask additional questions. Participants who did not report change of practice reported that it was mainly because of the lack of opportunities in their practices with, for instance, the above-average older population. Nonetheless, these participants reported that even after 6 months of participating in the study, they knew how they could change their practice in the future when they came across a patient with ADHD:

I offer them extra support, give them extra time in appointments… There are certain questions I might ask now that I wouldn’t before.

P2

My threshold to refer people for assessment would be much, much lower now.

P12

Finally, many GPs reported impacts beyond their practice. These participants discussed how the course allowed them to identify ADHD among family members or individuals they know in other settings. The participants also often disseminated the course within their contacts and practice, broadening the impact of the course. Finally, participants also reported seeking further training as a result of taking part in this course. Participants asked if we had more modules on similar topics available and also attended further training on ADHD and other developmental conditions as they wanted to learn more:

It helped me understand a little bit what was going on with my own son as well.

P18

It’s completely changed the way I view them, I’m much more sympathetic.

P14

I was able to pass on the learning to other doctors in our doctors meeting so. I’m hoping that will have impact not just on me but doctors at the surgery too.

P2

#### Surveys

A total of 21 participants (10/21, 48% females) responded to a brief web-based survey 6 months after taking part in the study. The responses were from a mix of participants who took part in the interviews (12/21, 58%) and those who did not (9/21, 42%).

These responses triangulated with the interview responses, and similar findings were observed. When asked whether the participants gained any knowledge on ADHD and if there was any difference in how they approached ADHD before and after their interaction with the course, 91% (20/21) of the participants agreed. When asked if the intervention had impacted their practice yet, 66% (14/21) said yes, 19% (4/21) said no, and 15% (3/21) said that the intervention had not yet impacted their practice.

When asked to give an example of how it changed their practice, GPs mentioned similar topics to the ones in the interviews, including an increase in referrals, more confidence in discussing and identifying ADHD, better use of assessment/screening tools, and better awareness and understanding of patients with ADHD.

Finally, when asked if the course impacted their attitude toward patients with or at risk for ADHD, 19% (4/21) of the participants reported no changes. Participants who reported changes in attitude mentioned increased empathy, better understanding, increased awareness of the positive impact of a diagnosis, and the importance of quick referrals as well as increased confidence. A decrease in common ADHD stigma such as bad parenting and misunderstanding that it only happens in childhood were also mentioned.

Survey responses from the group of GPs who did not take part in the interviews triangulated with the interview themes. In reporting the personal impact that the course had, GPs felt that it did change their attitude and knowledge of ADHD:

Better understanding of impact on individual and the support they need.

P14

I am more sympathetic to parents.

P19

GPs also reported a wide impact in their change of practice:

I have increased my referral to adult ADHD specifically rather than to psychological therapies.

P21

I saw a young boy the day after the training and It was very useful to know what questions to ask.

P8

## Discussion

### Principal Findings

With the aim of understanding the potential clinical utility of a web-based psychoeducation program aimed at improving GPs’ knowledge of ADHD, we conducted a pilot RCT and demonstrated that the intervention was potentially efficacious, with GPs reporting an increase in knowledge of ADHD, combined with a change in attitude, reduction in misconceptions, change in practice, and excellent reported levels of acceptability. Previous studies [9] have demonstrated that some of the major barriers in GPs’ understanding and management of ADHD reflect their lack of training and knowledge and the presence of misconceptions. This study has shown that a short web-based education program can be easily implemented and that it can address these gaps while also impacting practice. This study (with over 115/170, 67.6%) of GPs having never received any training on ADHD) and others [37,38] have highlighted the lack of initial GP training on ADHD. No difference was observed between participants who had and had not received previous ADHD training, indicating that the present training is ad hoc. Therefore, this intervention is timely in addressing these gaps.

The findings also highlight positive feedback on the usability and implementation of the intervention tool. Participants enjoyed taking part in the intervention and found it useful. A few participants reached out personally to the researchers to enquire about whether the tool could be shared with colleagues and GP trainees in their practice as they found it highly informative. None of the participants could think of anything that they felt was missing or that could have been changed. The usability of the web-based resource was initially investigated in a small pilot study [26], and the findings from this RCT confirm that the web-based resource is ready to be used as it is and that no further adjustment needs to be made. The findings from the interview also triangulate our previous findings of barriers in ADHD services in primary care, such as lack of appropriate services and lack of training [10]. GPs acknowledged that the lack of training on ADHD prompted them to do this intervention in the first place; although the increase in knowledge was useful, the lack of services to refer to, especially for adults, was frustrating. In contrast to findings from previous studies on the misconceptions and attitudes of GPs [31], our findings showed fewer misconceptions and stigmatizing views expressed by GPs. The intervention did address some of these; however, we found that at baseline, GPs were much less prone to stigmas than previously reported.

Few studies have investigated the implementation of web-based interventions for GPs. This study contributes to the body of work investigating methods of increasing the awareness of specific disorders among GPs [15] and providing accessible web-based educational programs. As GP training on ADHD is limited and no other targeted web-based education resource exists on the topic, this study addresses a vital gap. Piloting is important as it permits valuable methodological lessons to be learned. Although many pilot RCTs struggle to establish statistically significant results often because of small sample sizes [39-41], this study indicates the potential efficacy of the intervention, despite the limited sample size.

The coproduction approach [26] taken in developing the design and format of the web-based resource offers many strengths to this study. The resource is optimal for GPs as it is time limited, easily accessible, and freely available, minimizing the costs and time of the GPs in accessing training. Despite previous research highlighting difficulties with recruiting GPs as research participants [22,42,43], this study had no difficulty with recruitment. On the contrary, recruitment was very fast and had to close after only 2 weeks. This could indicate a high interest in the topic or the strong need for training on ADHD. Alternatively, and similar to the advice given in recent studies, the presence of monetary and nonmonetary (CPD certificate) incentives [44] and regular reminders [42] might have also contributed to the success in recruiting for this study.

### Limitations

A few limitations can also be highlighted in this study. The sample was not balanced across genders and included a high proportion of women (103/170, 60.6%). A recent report from the England General Medical Council [45] suggests that this is representative of part-time but not full-time permanent contracts in the NHS (only 4004/11,441, 34.99% of GPs on full-time permanent contracts are female, against 5008/8341, 60.04% part-time). Unfortunately, we did not collect information on whether the participants worked part-time or full-time, and this finding might imply that participants were more likely to participate if they worked part-time and therefore had more time to complete the study. It is also important to highlight that this study took place in England and is therefore specific to the British health care system where GPs acting as gatekeepers and providing referrals to secondary care services for diagnosis and treatment are the norm. Therefore, recommendations presented in the web-based resource as well as the design for this study reflected this specific system and might not apply to countries using a different approach.

Limitations also arose from a lack of methodological rigor that had to be adopted for pragmatic reasons. First, the assessor was not blinded to the study, and although the participants were blinded, they could potentially guess their group allocation. Although this can be an issue in reporting the rigor of this pilot RCT, the findings indicate that this had limited impact and are still worthy of a full RCT. Second, as a pilot efficacy RCT, there was no formal power calculation to inform the sample size. Nevertheless, the achieved sample size was sufficient to demonstrate postintervention differences between arms. Third, because of the format of the web-based intervention, randomization had to be performed before baseline, which is not common practice. Conducting randomization after baseline questionnaires would have added another step to the study, asking the GPs to spare time for more than one session, and therefore was believed to be likely to increase attrition. Sending specific links to either control or intervention groups so that GPs could complete questionnaires at T1 and T2 in 1 session seemed preferable to maximize the completion rate. However, despite clear instructions, less than 50% of the GPs completed in 1 session, and therefore randomization after baseline might not have had a significant impact on attrition. A total of 18 participants had to be excluded from the analyses after taking longer than 48 hours between the 2 time points. Therefore, completion in 1 session, although ideal for this study, seemed unfeasible for most participants.

Although a significant number of participants who completed consent forms did not take part in the study (60/231, 25.9%), this dropout can be explained by multiple factors. Recruitment in general practice is complex. Often practices are recruited for studies, and a selective number of GPs take part. Either practices or practice managers will express an interest for the participation of their practice. A couple of participants who were excluded as they had previously taken part in our pilot study explained that they provided consent on behalf of their practices. In the future, the expression of interest and consent for individuals versus practice will be made clearer. Attrition rates were moderate at 23.3% (40/171) between T1 and T3. However, the attrition rate between T2 and T3 was very low (2/133, 1.5%). A few retention strategies such as weekly reminders with clear deadlines and reinforcing the incentives were put in place, which seemed to minimize the attrition rate compared with average attrition rates of RCT [46,47].

Future research should address the methodological issues arising from this study. However, although it impacted attrition and exclusion rates, these issues do not seem to have impacted the findings for this study per se. Some changes in practice were observed; however, because of the time restriction for this study (6 months), we were unable to fully assess this impact over time. Future research should include a longitudinal assessment to explore whether changes in knowledge, attitude, and practice are retained over a longer period. Exploring the impact of this resource on other health care professionals, such as primary care nurses or secondary care professionals, would also allow for broader impacts of this intervention to be investigated. Finally, although qualitative data on change of practice were obtained in this study, assessing the impact on the number and quality of referrals was not possible within the context of this study. Future studies should include an assessment of referral or observational components to gauge changes in practice more directly.

### Conclusions

This pilot RCT was successful in answering the hypotheses that a short web-based psychoeducation program would increase the awareness, knowledge, and attitude of GPs toward ADHD while also changing their practice. These findings need to be interpreted with caution, as this is the only study investigating the efficiency of this web-based intervention, and further studies are needed to replicate these findings. These findings however highlight potential significant clinical impacts on the care and policies for patients. Through improved GP understanding and knowledge, patients should receive more timely access to care, reducing the long-term impacts of untreated and undiagnosed ADHD. This web-based resource has already been adopted by the RCGP, which will impact the learning and awareness of many GPs beyond this study, having a broader impact on practice and potentially influencing commissioning decisions once the importance of training GPs on ADHD has been recognized.

## Appendix

Multimedia Appendix 1 Participants’ demographics.

Multimedia Appendix 2 Descriptive statistics of the knowledge, misconceptions, and confidence scores of the Knowledge of Attention Deficit Disorders Scale for the 2 groups at 3 different time points.

Multimedia Appendix 3 Descriptive statistics of the knowledge scores of the Knowledge of Attention Deficit Disorders Scale on 3 subscales at 3 different time points.

Multimedia Appendix 4 CONSORT e-HEALTH Checklist V1.6.2.

## Abbreviations

ADHD: attention-deficit/hyperactivity disorder
ANCOVA: analysis of covariance
CPD: continuing professional development
CRN: clinical research network
GP: general practitioner
KADDS: Knowledge of Attention Deficit Disorders Scale
NHS: National Health Service
RCGP: Royal College of General Practitioner
RCT: randomized controlled trial
T1: time point 1
T2: time point 2
T3: time point 3
