# Supplementary material for: Evaluation of a Web-Based ADHD Awareness Training in Primary Care: Pilot Randomized Controlled Trial With Nested Interviews
Source: JMIR Med Educ. 2020 Dec 11;6(2):e19871. doi: 10.2196/19871 (PMC7762685; doi:10.2196/19871)
Supplement: Multimedia Appendix 1 [file mededu_v6i2e19871_app1.docx]

**Multimedia Appendix 1.** Participants’ demographics.

*Demographic*

No significant differences were found in KADDs scores at baseline between male (M=16.80, n=42) and female participants (M=16.76, n=67), U=1,435, p= .86 as well as no difference in confidence scores between male (M=4.7, n=42) and female participants (M=4.4, n=67), U=1,284, p= .43.

There was no significant difference in the KADDs scores at baseline between age groups (GP1, n=31: 25-35yrs, Gp2, n=41: 36-45yrs, Gp3, n=29: 46-55yrs, Gp4, n=8: 56-65yrs), Χ^2^ (3, n=109) = 3.65, p= .32 as well as no difference in confidence scores between age groups, Χ^2^ (3, n=109) = 5.95, p= .11.

Prior ADHD training had no significant effect on the KADDs scores at baseline between participants who had received prior training on ADHD (M=17.5, n=26) and those who had not (M=16.5, n=80), U=900, p= .30. A significant difference in self-rated confidence was observed between the groups who had received training on ADHD (M=5.4, n=26) or not (M=4.25, n=80) U= 640, p= .003. However, there was no significant effect of KADDS confidence score on those who had received prior training (M= 6.53, n=26) and those who had not (M=7.39, n=80) U=1,151, p= .41

The relationship between scores and GPs years of practice was also investigated. There was no correlation between years of practice and KADDS scores, r= .13, n=109, p= .154 and between years of practice and confidence scores, r= .17, n=109, p= .06

The relationship between scores and the number of ADHD cases GPs had identified was also explored. There was no correlation between identified cases and KADDS scores, r= .06, n=103, p= .49 and confidence scores, r= .0.2, n=103, p= .80

The relationship between scores and the number of suspected cases of ADHD in the GPs’ practices and the number of ADHD diagnoses in the GPs’ practices was also investigated. There was no correlation between KADDS scores and suspected cases of ADHD, r= .01, n=101, p= .89 and between KADDS scores and diagnosed cases, r= .17, n=71, p= .14 or confidence scores and suspected cases of ADHD or confidence scores and ADHD diagnoses.

No relationship between confidence scores and suspected cases of ADHD was observed, as well as between confidence scores and ADHD diagnosis.
