# Supplementary material for: Evaluation of a Web-Based ADHD Awareness Training in Primary Care: Pilot Randomized Controlled Trial With Nested Interviews
Source: JMIR Med Educ. 2020 Dec 11;6(2):e19871. doi: 10.2196/19871 (PMC7762685; doi:10.2196/19871)
Supplement: Multimedia Appendix 2 [file mededu_v6i2e19871_app2.docx]

**Multimedia appendix 2:** Supplementary Table 1- descriptive statistics of the KADDS knowledge, misconceptions and confidence scores^1^ for the two groups at the three different time points.

|  | | | KADDS Knowledge | | KADDS  Misconceptions | | KADDS Confidence | | Self-rated confidence | |
| --- | --- | --- | --- | --- | --- | --- | --- | --- | --- | --- |
|  |  |  | M | SD | M | SD | M | SD | M | SD |
| Control group | | T1  T2  T3 | 16.82  17.23  17.13 | 5.15  5.18  5.02 | 1.82  2.05  2.24 | 1.78  1.62  1.77 | 7.15  6.64  6.69 | 6.07  5.99  5.97 | 4.40  4.57  4.88 | 1.66  1.67  1.72 |
| Intervention group | T1  T2  T3 | | 16.65  23.71  22.96 | 3.88  2.00  2.13 | 2.16  1.54  1.70 | 2.20  1.55  1.65 | 7.12  0.73  1.22 | 4.30  1.35  1.71 | 4.66  7.40  7.36 | 1.70  1.05  0.89 |
