# Supplementary material for: Evaluation of a Web-Based ADHD Awareness Training in Primary Care: Pilot Randomized Controlled Trial With Nested Interviews
Source: JMIR Med Educ. 2020 Dec 11;6(2):e19871. doi: 10.2196/19871 (PMC7762685; doi:10.2196/19871)
Supplement: Multimedia Appendix 3 [file mededu_v6i2e19871_app3.docx]

Multimedia appendix 3 - Common attitudes and beliefs about ADHD in General practice.

|  | | | Control (n=52) | | | Intervention (n=57) | | |
| --- | --- | --- | --- | --- | --- | --- | --- | --- |
|  | | | No | Yes | Don’t know | No | Yes | Don’t know |
| Children with ADHD misbehave because they don’t follow rules | | T1  T2  T3 | 48 (92%)  49 (94%)  46 (88%) | 1 (2%)  1 (2%)  4 (7%) | 3 (6%)  2 (4%)  2 (4%) | 44 (77%)  51 (89%)  52 (91%) | 8 (14%)  5 (8%)  4 (7%) | 5 (8%)  1 (2%)  1 (2%) |
| Media coverage affects people’s conception of ADHD and its treatment | T1  T2  T3 | | 0  1(2%)  1 (2%) | 52 (100%)  50 (96%)  50 (96%) | 0  1 (2%)  1 (2%) | 1 (2%)  2 (4%)  2 (4%) | 55 (96%)  55 (96%)  55 (96%) | 1 (2%)  0  0 |
| Most children with ADHD try to control themselves | T1  T2  T3 | | 7 (13%)  7 (13%)  9 (17%) | 31 (60%)  34 (65%)  32 (61%) | 14 (27%)  11 (22%)  11 (22%) | 14 (24%)  13 (23%)  10 (17%) | 21 (37%)  36 (63%)  42 (74%) | 22 (38%)  8 (14%)  5 (8%) |
| Parent seek ADHD diagnosis as an excuse for their child’s bad behaviour | T1  T2  T3 | | 29 (55%)  28 (53%)  32 (62%) | 15 (29%)  14 (27%)  14 (27%) | 8 (15%)  10 (20%)  6 (11%) | 26 (45%)  46 (81%)  48 (84%) | 23 (40%)  8 (14%)  5 (8%) | 8 (14%)  3 (5%)  4 (7%) |
| Children’s inattentiveness is caused by unwillingness to please | T1  T2  T3 | | 49 (94%)  50 (96%)  51 (98%) | 0  0  0 | 3 (6%)  2 (4%)  1 (2%) | 54 (94%)  57 (100%)  55 (96%) | 2 (3.5%)  0  2 (4%) | 1 (2%)  0  0 |
| Parents primarily seek ADHD diagnosis to claim benefits | T1  T2  T3 | | 47 (91%)  44 (85%)  43 (83%) | 1 (2%)  1 (2%)  2 (4%) | 4 (7%)  7 (13%)  7 (13%) | 51 (89%)  56 (98%)  55 (96%) | 3 (5%)  1 (2%)  1 (2%) | 3 (5%)  0  1 (2%) |
| An ADHD diagnosis removes stigma of bad parenting | T1  T2  T3 | | 20 (38%)  21 (40%)  20 (38%) | 23 (45%)  20 (38%)  22 (42%) | 9 (17%)  11 (22%)  10 (20%) | 26 (45%)  26 (45%)  28 (49%) | 25 (44%)  27 (47%)  27 (47%) | 6 (10%)  4 (7%)  2 (4%) |
| An ADHD diagnosis is helpful for an individual | T1  T2  T3 | | 2 (4%)  0  1 (2%) | 41 (79%)  42 (80%)  42 (80%) | 9 (17%)  10 (20%)  9 (17%) | 4 (7%)  2 (4%)  2 (4%) | 49 (86%)  55 (96%)  53 (92%) | 4 (7%)  0  2 (4%) |
| An ADHD diagnosis is stigmatising for an individual | T1  T2  T3 | | 14 (33%)  11 (21%)  16 | 27 (52%)  26 (50%)  26 (50%) | 11 (21%)  15 (29%)  10 (20%) | 21 (37%)  30 (53%)  25 (44%) | 29 (51%)  21 (37%)  26 (45%) | 7 (12%)  6 (10%)  6 (10%) |
| ADHD diagnosis relieves families from stress and support problem solving | T1  T2  T3 | | 6 (11%)  2 (4%)  4 (7%) | 39 (76%)  42 (81%)  38 (73%) | 7 (13%)  8 (15%)  10 (20%) | 8 (14%)  4 (7%)  3 (5%) | 42 (74%)  50 (88%)  51 (89%) | 7 (12%)  3 (5%)  3 (5%) |
| Do you believe ADHD is a valid diagnosis? | T1  T2  T3 | | 0  0  0 | 46 (89%)  49 (94%)  50 (96%) | 6 (11%)  3 (6%)  1 (2%) | 2 (4%)  1 (2%)  1 (2%) | 50 (88%)  55 (96%)  56 (98%) | 3 (5%)  0  0 |
| Do you believe ADHD is society’s excuse for badly behaved children | T1  T2  T3 | | 42 (81%)  42 (81%)  43 (83%) | 1 (2%)  2 (4%)  2 (4%) | 9 (17%)  8 (15%)  7 (13%) | 46 (81%)  54 (94%)  52 (91%) | 2 (4%)  2 (4%)  1 (2%) | 9 (15%)  1 (2%)  3 (5%) |

| The numbers represents the participants’ response for each item |  |  |  |
| --- | --- | --- | --- |
