# Supplementary material for: Evaluation of a Web-Based ADHD Awareness Training in Primary Care: Pilot Randomized Controlled Trial With Nested Interviews
Source: JMIR Med Educ. 2020 Dec 11;6(2):e19871. doi: 10.2196/19871 (PMC7762685; doi:10.2196/19871)
Supplement: Multimedia Appendix 4 [file mededu_v6i2e19871_app4.pdf]

CONSORT-EHEALTH Checklist V1.6.2 Report	Manuscript Number	19871
**(based on CONSORT-EHEALTH V1.6), available at [http://tinyurl.com/consort-ehealth-v1-6].**		
**Date completed**		
6/23/2020 5:05:55		
**by**		
Blandine French		
Assessing the efficacy of online ADHD awareness training in primary care: Pilot randomised control trial evaluation with nested qualitative interviews		
TITLE		
**1a-i) Identify the mode of delivery in the title**		
yes "online ADHD awareness training". This intervention ran on mobile, PC or tablet so online was felt to be the most appropriate term		
**1a-ii) Non-web-based components or important co-interventions in title**		
yes " Pilot randomised control trial evaluation with nested qualitative interviews"		
**1a-iii) Primary condition or target group in the title**		
Yes "Assessing the efficacy of online ADHD awareness training in primary care: "		
ABSTRACT		
**1b-i) Key features/functionalities/components of the intervention and comparator in the METHODS section of the ABSTRACT**		
yes "participants were randomised to either an intervention control or an awareness training intervention"		
**1b-ii) Level of human involvement in the METHODS section of the ABSTRACT**		
Yes "assessed the efficacy of an online psychoeducation program"		
**1b-iii) Open vs. closed, web-based (self-assessment) vs. face-to-face assessments in the METHODS section of the ABSTRACT**		
No		
**1b-iv) RESULTS section in abstract must contain use data**		
yes " A total of 109 GPs' responses were included in the analysis" "completed questionnaires on ADHD knowledge, confidence and attitude at three time-points "		
**1b-v) CONCLUSIONS/DISCUSSION in abstract for negative trials**		
INTRODUCTION		
**2a-i) Problem and the type of system/solution**		
yes "One perceived barrier to GPs attending and participating in training may be having to travel long distances to attend training sessions, which may be particularly burdensome for GPs serving in remote communities [22]. The development of online training may go some way in reducing this barrier, offering GPs easily accessible training at a time and place that fits their busy schedules. The use of online training by healthcare professionals has significantly increased in recent years [23–25]. Online training is an efficacious mode of delivery, with a recent review demonstrating that online continuing medical education improves knowledge and changes GPs' practice [22]. To our knowledge, no studies have been published on ADHD online psychoeducation programs developed for GPs and data are lacking on the efficacy of ADHD training programs for GPs."		
**2a-ii) Scientific background, rationale: What is known about the (type of) system**		
yes "Gaining a diagnosis of ADHD is important for access to appropriate treatment and minimising the long-term impacts of ADHD. However, in many countries, ADHD is underdiagnosed and undertreated [5–7]." "GPs do not always readily recognise ADHD symptoms; with many reporting low confidence, limited knowledge and strong misconceptions about the disorder [9–11]. "		
**Does your paper address CONSORT subitem 2b?**		
yes "the present study aims to obtain preliminary findings on the effect of the "Understanding ADHD in primary care" online program on GPs' ADHD knowledge, attitudes, misconceptions and change of practice, to determine whether a future definitive randomised controlled trial (RCT) should be conducted"		
METHODS		
**3a) CONSORT: Description of trial design (such as parallel, factorial) including allocation ratio**		
yes "This parallel-group, single-blind randomised control trial "		
**3b) CONSORT: Important changes to methods after trial commencement (such as eligibility criteria), with reasons**		
Not applicable in this pilot trial. The process of refining the intervention is published elsewhere		
**3b-i) Bug fixes, Downtimes, Content Changes**		
Not applicable, the intervention had no bugs and a usability study addressed any changes to the content (currently submitted for publication)		
**4a) CONSORT: Eligibility criteria for participants**		
yes "GPs and GP trainees practicing in England were included, the only exclusion criteria was having taken part in a previous usability study"		
**4a-i) Computer / Internet literacy**		
No, this study included GPs, who have to use a compuyter as part of their job therefore it didn't feel necessary to include this as a criteria		
**4a-ii) Open vs. closed, web-based vs. face-to-face assessments:**		
yes "were recruited from multiple sites across England and responded to invitation emails from local clinical research networks, (CRNs) sent out via their practice"		
**4a-iii) Information giving during recruitment**		
Yes "Participants who expressed interest were sent a link to an online consent form and information sheet".		
**4b) CONSORT: Settings and locations where the data were collected**		
yes "Twelve out of 15 English CRNs distributed the study invitation to hundreds of practices"		
**4b-i) Report if outcomes were (self-)assessed through online questionnaires**		
yes "These questionnaires were administered at online three time-points"		
**4b-ii) Report how institutional affiliations are displayed**		
yes "The online resource was delivered using a University of Nottingham server and built with an open-source learning management system. "		
**5) CONSORT: Describe the interventions for each group with sufficient details to allow replication, including how and when they were actually administered**		
**5-i) Mention names, credential, affiliations of the developers, sponsors, and owners**		
yes "Prof. Daley reports grants, personal fees and non-financial support from Shire/Takeda, personal fees and non-financial support from Medice, personal fees and non-financial support from Eli Lilly, non-financial support from QbTech, outside the submitted work. Mrs French reports personal fees and non-financial support from Shire/Takeda. Prof. Sayal was a member of the NICE ADHD Guideline Development Group (NG87), Dr Hall and Dr Perez Vallejos report no conflicts of interest."		
**5-ii) Describe the history/development process**		
yes "The online intervention was developed by the researchers following a strict development process and its usability has been previously assessed [26]."		
**5-iii) Revisions and updating**		
Not applicable for this study as changes were made after usability study, " No changes were made to either interventions during the trial."		
**5-iv) Quality assurance methods**		
Not applicaqble as it was a pilot study		
**5-v) Ensure replicability by publishing the source code, and/or providing screenshots/screen-capture video, and/or providing flowcharts of the algorithms used**		
Not applicable		
**5-vi) Digital preservation**		
yes ""ADHD toolkit" including downloadable screening tools, strategies or useful websites [28]"		
**5-vii) Access**		
yes "Post randomisation, participants were then sent a link to the online resource of their allocated group. "An inconvenience allowance and CPD certificate from the Royal College of GPs (RCGP) were attributed to the participants upon completion of the questionnaire at T3"		
**5-viii) Mode of delivery, features/functionalities/components of the intervention and comparator, and the theoretical framework**		

Yes "The online resource was delivered using a University of Nottingham server and built with an open-source learning management system. Further details on the intervention development are reported elsewhere [26]. The complete online resource consisted of two 20-minute modules undertaken sequentially. The two modules followed the same format with text on the left side of the screen and interactive activities on the right. The activities included patient testimonies, drag and drop games, specialist videos and pictures.<br>Module 1: Called "Understanding ADHD" - included the heterogeneous nature of ADHD; brief description of ADHD epidemiology, neuroscience; ADHD symptoms, comorbidity, risks and common misconceptions.<br>Module 2: Called "The role of the GP" - introduced the GP's role in ADHD diagnosis and treatment pathways; identification of ADHD and subsequent treatment options; GP's gatekeeping role and the pathway to care in the UK and an "ADHD toolkit" including downloadable screening tools, strategies or useful websites [28].<br>Control online resource<br>Participants allocated to the control group watched an online 30-minute video about the University of Nottingham, Institute of Mental Health [29]. No information related to ADHD was provided during this video."		
**5-ix) Describe use parameters**		
yes "All elements of the intervention were compulsory, and participants had to take part in all the stages to contribute to the study." "the complete online resource consisted of two 20-minute modules undertaken sequentially. " Control:"Participants allocated to the control group watched an online 30-minute video about "		
**5-x) Clarify the level of human involvement**		
No human support was offered "Participants wishing to take part signed an online consent form, upon receiving consent, they were randomly allocated to intervention or control group. Post randomisation, participants were then sent a link to the online resource of their allocated group"		
**5-xi) Report any prompts/reminders used**		
yes "Weekly reminders were sent via email for four weeks by the researcher. "		
**5-xii) Describe any co-interventions (incl. training/support)**		
Not applicable		
**6a) CONSORT: Completely defined pre-specified primary and secondary outcome measures, including how and when they were assessed**		
yes "The primary outcome was: change in GPs' knowledge assessed by the KADDS ." "Secondary outcomes: Changes in knowledge (assessed via KADDS questionnaire) were re-assessed two weeks after completing the intervention (time 3; T3). Analyses of subscales of the KADDS questionnaire were also explored. Further secondary outcomes included:<br>GPs' confidence in ADHD: Change in confidence was explored through a self-rated visual analogue scale (1 being low, 10 being high) assessing GPs' confidence in their knowledge of ADHD. "		
**6a-i) Online questionnaires: describe if they were validated for online use and apply CHERRIES items to describe how the questionnaires were designed/deployed**		
No		
**6a-ii) Describe whether and how "use" (including intensity of use/dosage) was defined/measured/monitored**		
yes, use was described as completion of questionnaire "Participants who did not complete all time points were also excluded from the completer analysis "		
**6a-iii) Describe whether, how, and when qualitative feedback from participants was obtained**		
yes "A 4-item open questionnaire was sent to all 56 participants from the intervention arm who consented, which assessed changes in practice and approaches, six months post-intervention.<br>Secondary outcomes also included exploration of attitudes towards ADHD and long term self-reported change in practice. Change in practice was assessed through semi-structured interviews and a short survey. The interview schedule included questions about the intervention and the impact it had on GPs' attitude and practice."<br>"		
**6b) CONSORT: Any changes to trial outcomes after the trial commenced, with reasons**		
yes "Twelve out of 15 English CRNs distributed the study invitation to hundreds of practices"		
**7a) CONSORT: How sample size was determined**		
**7a-i) Describe whether and how expected attrition was taken into account when calculating the sample size**		
Not applicable as this was a pilot sudy		
**7b) CONSORT: When applicable, explanation of any interim analyses and stopping guidelines**		
yes "The primary outcome was: change in GPs' knowledge assessed by the KADDS ." "Secondary outcomes: Changes in knowledge (assessed via KADDS questionnaire) were re-assessed two weeks after completing the intervention (time 3; T3). Analyses of subscales of the KADDS questionnaire were also explored. Further secondary outcomes included:<br>GPs' confidence in ADHD: Change in confidence was explored through a self-rated visual analogue scale (1 being low, 10 being high) assessing GPs' confidence in their knowledge of ADHD. "		
**8a) CONSORT: Method used to generate the random allocation sequence**		
yes "Randomisation was initiated by the primary author and performed online through a randomisation website [33] in batches of 20"		
**8b) CONSORT: Type of randomisation; details of any restriction (such as blocking and block size)**		
yes "Randomisation was initiated by the primary author and performed online through a randomisation website [33] in batches of 20"		
**9) CONSORT: Mechanism used to implement the random allocation sequence (such as sequentially numbered containers), describing any steps taken to conceal the sequence until interventions were assigned**		
yes "Randomisation was initiated by the primary author and performed online through a randomisation website [33] in batches of 20"		
**10) CONSORT: Who generated the random allocation sequence, who enrolled participants, and who assigned participants to interventions**		
yes "Randomisation was initiated by the primary author and performed online through a randomisation website [33] in batches of 20"		
**11a) CONSORT: Blinding - If done, who was blinded after assignment to interventions (for example, participants, care providers, those assessing outcomes) and how**		
**11a-i) Specify who was blinded, and who wasn't**		
yes "The outcome assessor and interviewer were not blind to group allocation.<br>Due to the nature of the study, participants were blind to study arm but may have been able to guess their arm once they started the study."		
**11a-ii) Discuss e.g., whether participants knew which intervention was the "intervention of interest" and which one was the "comparator"**		
"Due to the nature of the study, participants were blind to study arm but may have been able to guess their arm once they started the study. "		
**11b) CONSORT: If relevant, description of the similarity of interventions**		
There were no similarities between the control and the intervention " No information related to ADHD was provided during this video. "		
**12a) CONSORT: Statistical methods used to compare groups for primary and secondary outcomes**		
yes "Preliminary checks were conducted to ensure that there was no violation of the assumptions of normality, linearity, homogeneity of variances and reliable measurement of the covariate. A significant Kolmogorov-Smirnov test showed that the data was not normally distributed, therefore non-parametric tests were used. Mann-Whitney U and Kruskal-Wallis tests were used to explore demographic differences between trial arms. A Spearman correlation was used to determine the relationship between KADDS and confidence scores. KADDS questionnaire scores were the primary outcome at T2, self-ratings of confidence were also explored and both variables were analysed using analyses of covariance, with T1 entered as the covariate as ANCOVA is robust to violation of the non-parametric assumption with moderate to large sample sizes, greater than 15 cases per cell [34]. Outcome at T3 was also explored using the same analytical approach. Both total and subscale scores of the KADDS were explored. "		
**12a-i) Imputation techniques to deal with attrition / missing values**		
No "Participants who did not complete all time points were also excluded from the completer analysis as an intention-to-treat analysis was not possible due to randomisation before baseline."		
**12b) CONSORT: Methods for additional analyses, such as subgroup analyses and adjusted analyses**		
yes "The analytic strategy for this study was based on thematic analysis [35] enhanced by the principles of grounded theory [36]. Themes and subthemes were identified using an adapted approach of Braun and Clarke's [20] six stage process. The analytic process began by transcribing each interview verbatim, shortly after being conducted. Following this process, the lead investigator first familiarized herself with the interviews and made notes in a diary of preliminary thoughts on the content of the interviews. From this, preliminary codes were identified in a coding manual that were then collated and combined to be classified into broader themes using constant comparative analysis both within and between transcripts. Finally, as the analysis evolved, these broader themes were reviewed and refined and generated the final themes proposed. Ongoing analysis allowed for a clear definition of the final themes.<br>Themes were finally reviewed by a second researcher (EPV) to ensure that they mapped on to the original transcripts. Inter-rater reliability was tested on a small proportion (10%) of the transcripts' themes. The results were validated collectively as a team, and any discrepancies were discussed and reconciled. The survey responses were reported descriptively and used to triangulate the responses from the interview. "		
RESULTS		
**13a) CONSORT: For each group, the numbers of participants who were randomly assigned, received intended treatment, and were analysed for the primary outcome**		

yes "Therefore, 221 participants were randomised, 111 in the intervention group and 110 in the control group. Post randomisation 51 GPs (27 intervention and 23 control) did not respond to the invitation to start the study. Figure 1 shows the numbers lost to follow-up at each point. Upon answering the baseline questionnaire, 37 GPs did not complete the post questionnaire (17 intervention and 20 control) at time 2 and two GPs (one intervention and one control) at time 3. 170 trainees or fully qualified GPs (103 Female: 60.5%, 6 GP trainees: 3.5%) completed Time 1, 133 completed T1 and T2 (84 Females: 63.1%, 5 GP trainees: 3.7%) and 131 (82 Females: 62.5%, 5 GP trainees: 3.8%) completed all three time points.<br>Twenty-two participants were excluded from the analyses following protocol violations. "		
**13b) CONSORT: For each group, losses and exclusions after randomisation, together with reasons**		
yes ". Post randomisation 51 GPs (27 intervention and 23 control) did not respond to the invitation to start the study. " Please see figure 1		
**13b-i) Attrition diagram**		
yes "Figure 1- Consort flow chart of the pilot RCT.", see page 8		
**14a) CONSORT: Dates defining the periods of recruitment and follow-up**		
yes "Participants were recruited between the 10th of July 2019 and the 23rd of August 2019 and were followed up until the 30th of October 2019. "		
**14a-i) Indicate if critical "secular events" fell into the study period**		
No as no seculiar events occured		
**14b) CONSORT: Why the trial ended or was stopped (early)**		
Yes, the trial ended with the completion of the final questionnaire " were followed up until the 30th of October 2019 when the trial ended"		
**15) CONSORT: A table showing baseline demographic and clinical characteristics for each group**		
yes "Table 1- Baseline characteristics "		
**15-i) Report demographics associated with digital divide issues**		
yes, as our sample were educated GPs, education and computer literacy were less relevant, Table 1 shows baseline characteristics of the sample		
**16a) CONSORT: For each group, number of participants (denominator) included in each analysis and whether the analysis was by original assigned groups**		
**16-i) Report multiple "denominators" and provide definitions**		
yes "A total of 231 GPs registered their interest in the study and consented to take part. Ten GPs did not meet eligibility criteria (See Figure 1) and were not enrolled in the trial.<br>Therefore, 221 participants were randomised, 111 in the intervention group and 110 in the control group. Post randomisation 51 GPs (27 intervention and 23 control) did not respond to the invitation to start the study. Figure 1 shows the numbers lost to follow-up at each point. Upon answering the baseline questionnaire, 37 GPs did not complete the post questionnaire (17 intervention and 20 control) at time 2 and two GPs (one intervention and one control) at time 3. 170 trainees or fully qualified GPs (103 Female: 60.5%, 6 GP trainees: 3.5%) completed Time 1, 133 completed T1 and T2 (84 Females: 63.1%, 5 GP trainees: 3.7%) and 131 (82 Females: 62.5%, 5 GP trainees: 3.8%) completed all three time points."		
**16-ii) Primary analysis should be intent-to-treat**		
No "Participants who did not complete all time points were also excluded from the completer analysis as an intention-to-treat analysis was not possible due to randomisation before baseline."		
**17a) CONSORT: For each primary and secondary outcome, results for each group, and the estimated effect size and its precision (such as 95% confidence interval)**		
No		
**17a-i) Presentation of process outcomes such as metrics of use and intensity of use**		
No, not applicable		
**17b) CONSORT: For binary outcomes, presentation of both absolute and relative effect sizes is recommended**		
No, Not applicable		
**18) CONSORT: Results of any other analyses performed, including subgroup analyses and adjusted analyses, distinguishing pre-specified from exploratory**		
yes, see information under heading "Interviews and surveys"		
**18-i) Subgroup analysis of comparing only users**		
Not applicable		
**19) CONSORT: All important harms or unintended effects in each group**		
no, no harm was anticipated as participants were NHS staff and not patients		
**19-i) Include privacy breaches, technical problems**		
no, this did not occur		
**19-ii) Include qualitative feedback from participants or observations from staff/researchers**		
Yes, interview and survey question is dedicated to this		
DISCUSSION		
**20) CONSORT: Trial limitations, addressing sources of potential bias, imprecision, multiplicity of analyses**		
**20-i) Typical limitations in ehealth trials**		
yes, the limitation section discusses all thes important limitation of ehealth trials		
**21) CONSORT: Generalisability (external validity, applicability) of the trial findings**		
**21-i) Generalizability to other populations**		
yes "Exploring the impact of this resource on other healthcare professionals such as primary care nurses or secondary care professionals would also allow for broader impacts of this intervention to be investigated."		
**21-ii) Discuss if there were elements in the RCT that would be different in a routine application setting**		
Yes, this was not addressed specifically but implied ease of use through the online aspect and easy accessibility fo this intervention " This study contributes to the body of work investigating methods of increasing GPs' awareness of specific disorders [15] and providing accessible online educational programs"		
**22) CONSORT: Interpretation consistent with results, balancing benefits and harms, and considering other relevant evidence**		
**22-i) Restate study questions and summarize the answers suggested by the data, starting with primary outcomes and process outcomes (use)**		
yes "With the aim of understanding the potential clinical utility of an online psychoeducation programme aimed at improving GPs' knowledge of ADHD, we conducted a pilot RCT and demonstrated that the intervention was potentially efficacious with GPs reporting an increase in knowledge of ADHD, combined with a change in attitude, decrease in misconceptions, change in practice and excellent reported levels of acceptability"		
**22-ii) Highlight unanswered new questions, suggest future research**		
yes "Future research should address methodological issues arising from this study. Yet, while it impacted attrition and exclusion rate, these issues do not seem to have impacted the findings for this study per se. Some changes in practice were observed however, due to the time restriction for this study (6 months), we were unable to fully assess this impact over time. Future research should include a longitudinal assessment to explore whether changes in knowledge, attitude and practice are retained over a longer period of time. Exploring the impact of this resource on other healthcare professionals such as primary care nurses or secondary care professionals would also allow for broader impacts of this intervention to be investigated. Finally, although qualitative data on change of practice was gained in this study, assessing the impact on the number and quality of referrals was not possible within the context of this study. Future studies should include an assessment of referral or observational components to gauge change in practice more directly. "		
Other information		
**23) CONSORT: Registration number and name of trial registry**		
yes "ISRCTN registry (ISRCTN45400501)"		
**24) CONSORT: Where the full trial protocol can be accessed, if available**		
N/A		
**25) CONSORT: Sources of funding and other support (such as supply of drugs), role of funders**		
yes "Dr Elvira Perez Vallejos acknowledges the financial support of the NIHR Nottingham Biomedical Research Centre. Blandine French acknowledges the financial support of the Economic and Social Research Council"		
**X26-i) Comment on ethics committee approval**		
yes "The study received ethical approval from the University of Nottingham, Faculty of Medicine and Health Sciences Research Ethics Committee (Ref: 19/HRA/1028, 20th of February 2019) and from the Nottinghamshire Healthcare NHS Foundation Trust R&D department (IRAS PROJECT ID 257567). "		
**x26-ii) Outline informed consent procedures**		
yes "Participants wishing to take part signed an online consent form"		
**X26-iii) Safety and security procedures**		
Not relevant, no patient data and no individual identifiable data was collected		
**X27-i) State the relation of the study team towards the system being evaluated**		
yes "The online non-commercial resource was delivered using a University of Nottingham server and built with an open-source learning management system." "The online intervention was developed by the researchers following a strict development process and "		
